# Maternal Service Coverage and Its Relationship To Health Information System Performance: A Linked Facility and Population-Based Survey in Ethiopia

**DOI:** 10.9745/GHSP-D-21-00688

**Published:** 2022-09-15

**Authors:** Abebaw Gebeyehu Worku, Hibret Alemu Tilahun, Hiwot Belay, Afrah Mohammedsanni, Naod Wendrad, Biruk Abate, Mesoud Mohammed, Mohammed Ahmed, Yakob Wondarad, Meskerem Abebaw, Wubshet Denboba, Frehiwot Mulugeta, Shemsedin Oumer, Amanuel Biru

**Affiliations:** a JSI Research and Training Institute, Inc., Ethiopia Data Use Partnership, Addis Ababa, Ethiopia.; b Ethiopia Ministry of Health, Addis Ababa, Ethiopia.

## Abstract

**Background:**

Studies in Ethiopia show an increasing trend in maternal health service use, such as having at least 4 visits of antenatal care (ANC4+) and skilled birth attendance (SBA). Improving the health information system (HIS) is an intervention that can improve service uptake and quality. We conducted a baseline study to measure current maternal service coverage, HIS performance status, and their relationship.

**Methods:**

We conducted a linked health facility-level and population-based survey from September 2020 to October 2020. The study covers all regions of Ethiopia. For the population-based survey, 3,016 mothers were included. Overall, 81 health posts, 71 health centers, and 15 hospitals were selected for the facility survey. A two-stage sampling procedure was applied to select target households. The study used modified Performance of Routine Information System Management tools for the facility survey and a structured questionnaire for the household survey. Multilevel logistic regression was employed to account for clustering and control for likely confounders.

**Results:**

Maternal service indicators, ANC4+ visits (54.0%), SBA (75.8%), postnatal care (70.6%), and cesarean delivery (9%) showed good service uptake. All data quality and use indicators showed lower performance compared to the national target of 90%. Maternal education and higher levels of wealth index were significantly and positively associated with all selected maternal service indicators. Longer distance from health facilities was significantly and negatively associated with SBA and the maternal care composite indicator. Among HIS-related indicators, availability of electronic HIS tools was significantly associated with maternal care composite indicator and ANC4+.

**Conclusions:**

Maternal service indicators showed promising performance. However, current HIS performance is suboptimal. Both service user and HIS-related factors were associated with maternal service uptake. Conducting similar research outside of the project sites will be helpful to have a wider understanding and better coverage.

## Abstract (precis)

Coverage for most maternal services showed promising performance. Improving the health information system performance can further improve maternal service uptake and quality.

## Teaser

**Key Findings:**

- Most of the maternal health care service coverage indicators, including 4 antenatal care visits, skilled birth attendance, cesarean delivery, and postnatal care, showed promising performance.
- Health information system (HIS) performance (data quality and data use) indicators had suboptimal performance compared with the national target.
- HIS-related variables including data accuracy and availability of digital tools were significantly associated with maternal service uptake.

**Key Implications:**

- Investing in a health information system is one of the key strategies to improve maternal service uptake. Interventions including the establishment of functional digital systems, availing guidelines, need-based trainings, and strengthening data quality control practices are crucial for better HIS.
- Addressing variations in terms of maternal service (access or quality) among health facilities is essential to maintain the current coverage and progressive trends of maternal service coverage indicators.

## INTRODUCTION

Access to high-quality maternal services by a skilled provider is a key factor in achieving better maternal and newborn health outcomes.^1^^–^^3^ During the past decade, Ethiopia has increased the number of health facilities and the number of trained, skilled health professionals offering maternal services, leading to an improvement in maternal service coverage across the country.^4^ For example, the percentage of births delivered by a skilled provider increased from 6% in 2005 to 50% in 2019.^5^ However, ensuring access to and maintaining a high quality of maternal health care services requires following up daily and making timely, informed decisions based on data, which depends on having an organized health information system at each health system level.^6^

To address the increased demands for data for decision making at the national and global levels, over the last several years, the Ministry of Health (MOH) in Ethiopia undertook an extensive reform of the national health information system (HIS).^7^^,^^8^ Guided by the Information Revolution roadmap,^9^ the Ethiopia Data Use Partnership (DUP) and local universities have been working with the MOH to address current HIS challenges on a national scale in both urban and rural settings, with the aim of transforming routine health information and improving the quality and use of data.

HIS at the health facility level is expected to be one of the key factors in improving maternal health care services. To better understand service readiness, service gaps, and service uptake, it is important to use the HIS to monitor service uptake and quality.^10^ This process can be done through the regular review of routine health data and conducting root cause analyses of issues when needed.^11^^,^^12^

There are a variety of factors that affect the utilization of maternal services. Client characteristics, such as education, residence, and socioeconomic status, consistently affect maternal service utilization.^13^^–^^15^ Supply side factors—including the availability of required equipment, supplies, medicine, trained health workers, and guidelines/standard operating procedures—can directly or indirectly affect service uptake and quality of service provision and are all noted gaps in health facilities across Ethiopia.^16^ For example, a study noted that important components of antenatal care (ANC) were not completed in health facilities, resulting in a low quality-of-care score.^17^^,^^18^

Hence, improving HIS performance (both in data quality and information use) while addressing HIS capacity and data management challenges at the health facility level is one of Ethiopia’s health sector priorities.^19^^,^^20^ HIS data quality is measured on 3 dimensions, including completeness (reporting, source document, and content), timeliness, and accuracy. Indices to measure information use include the use of information for performance review and evidence-based decision making, production of narrative analytical reports, health management information system (HMIS) quality improvement, planning/target setting, supervision, and data dissemination outside of the health sector.^21^ Technical factors, such as complexity of the reporting form/procedures, HIS design, computer software, and information technology complexity; behavioral factors, such as level of knowledge of the content of HIS forms, data quality checking skills, problem solving for HIS tasks, competence in HIS tasks, confidence levels for HIS tasks and motivation; and organizational factors, such as critical management functions and information needs, governance, planning, training, supervision, and finances, also affect HIS processes and interventions, which in turn affect HIS performance (in terms of data quality and use) and ultimately health systems performance and health outcomes. Some studies revealed how HIS interventions helped in improving data quality and information use for key public health programs like immunization.^22^

Improving HIS performance data quality and information use is one of Ethiopia’s health sector priorities.

Nonetheless, recent studies in Ethiopia showed that the quality of routine data gathered in the health system is poor,^23^ and the proportion of routine health information utilization for decision making is low.^24^ Moreover, no research has been conducted in Ethiopia designed to understand the relationship between HIS performance and health care service uptake/coverage. To address these gaps, the DUP launched a 2-arm quasi-experimental study to assess whether and how a multicomponent HIS strengthening intervention improves the quality and utilization of routine health data and whether exposure to it was associated with higher levels of utilization of essential maternal and child health care services.

In this article, we report on the results from the baseline data collection for this study. The objective of this analysis is to describe current maternal health care service coverage and HIS performance status and determine whether baseline utilization of maternal health care was associated with HIS indicators. The study findings will provide helpful insights on how the future intervention can best leverage available resources and maximize the impact of HIS strengthening on maternal health.

## METHODS

### Study Area and Design

To address current HIS challenges, the DUP provided focused and tailored HIS intervention packages in learning/demonstration sites (at least 1 woreda per region) to scale up best practices. Interventions include need-based trainings, supportive supervision, regular mentorship, and material support.

A 2-arm quasi-experimental study was implemented in intervention and comparison woredas in all regions of Ethiopia (Figure). Baseline and endline surveys were planned for both arms. A linked health facility-level and population-based survey were conducted at the baseline. The health facility-level survey focused on HIS performance (HIS intervention implementation, data quality, and use) in facilities that serve the selected kebele populations. A kebele is the lowest administrative unit having about 1,000 households while a woreda is an administrative unit comprising 25 to 30 kebeles, 5 to 6 health centers, and 1 district hospital. The household (population-based) survey focused on maternal service coverage data and was conducted in kebeles across selected woredas from September 21, 2020, to October 15, 2020.

**FIGURE.**
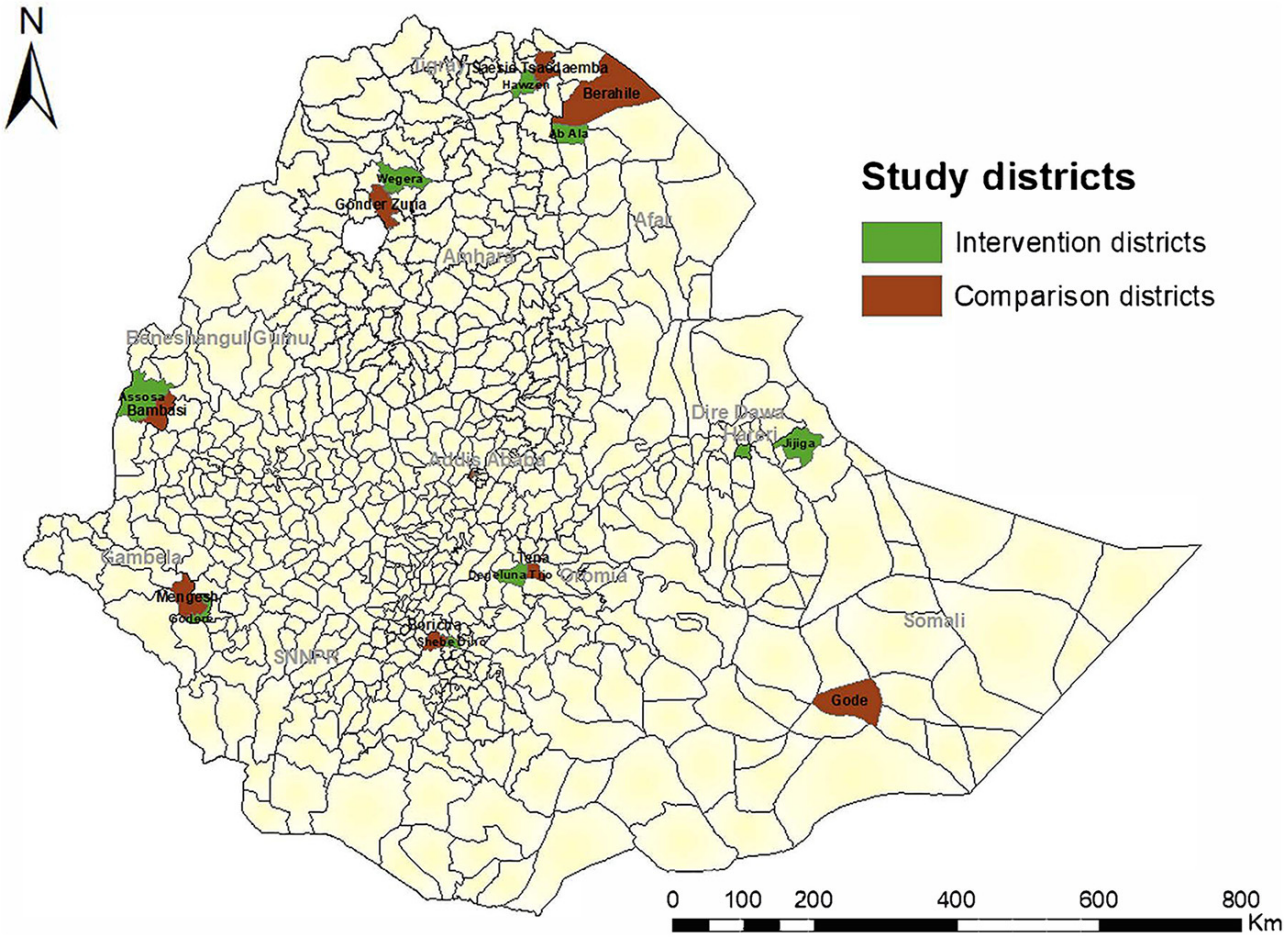
Map of Study Area Analyzing National Health Information System Strengthening Intervention, Ethiopia, 2020

### Target Population and Sampling

The target population for this study is women with children aged 12–23 months. A 2-stage sampling technique was applied to select the sample population. In the first stage, 102 kebeles were selected from 33 woredas. The selection is done from the list of kebeles per woreda. The number of selected kebeles was based on the population size of the regions. In the second stage, 29 targeted households were selected randomly from each kebele. The sampling frame of targeted households was prepared either using the health post data or census. A total of 3,016 households with targeted mothers (1 in each household) were included.

For the health facility survey, health posts and health centers providing services for the catchment population in selected kebeles were included in the sample, along with all hospitals in the 33 woredas. Overall, 81 health posts, 71 health centers, and 15 hospitals were selected for the facility survey. However, only 74 health facilities (70 health centers and 4 hospitals) data were linked with 3,016 households for the regression analysis.

### Data Collection and Procedures

A structured data collection tool was adapted from the Demographic and Health Survey (DHS) questionnaire^25^ and designed on SurveyCTO (software tool for data collection) to collect the population-based data. The household questionnaire was translated into 3 local languages, Amharic, Oromipha, and Tigrigna, and data collectors used the translated version during the interviews. Similarly, modified Performance Routine Information System Management (PRISM) tools,^26^ designed on SurveyCTO, were used for the health facility survey, which included key informant interviews, document review, and observation. A total of 54 data collectors (36 dedicated to households and 18 for health facilities) and 19 supervisors who had experience collecting survey data were deployed and used tablets to collect data. The survey team received an intensive 5-day training using a structured training manual. Before data collection began, the questionnaires and the SurveyCTO program were pretested. All the data collectors and supervisors were engaged in the pretesting translated tools.

### Data Analysis

Data use and data quality scores were generated from health facility data based on the PRISM tool. Mixed effect modeling (multilevel logistic regression) was employed to control the effect of clustering and potential confounders. Different data sets produced from the household and health facility survey were carefully cleaned and linked to perform regression analysis. The outcome variable (respondent maternal service utilization) depends on facility level and individual service users’ characteristics. The presence of 4 or more ANC (ANC4+) visits during pregnancy, skilled birth attendance (SBA), and maternal care composite indicator of maternal care services were selected outcome variables for the regression analysis (all from the household survey). The outcome variables have binary categories with “no” coded as “0” and “yes” coded as “1.”

To develop the composite indicator, 3 categories of care, antenatal, intrapartum, and postnatal, with equal weights were included. The development of composite indicators depends on interests to monitor programs in the continuum of care framework (no widely agreed-upon composite indicators). Some wanted to have the composite index including maternal and child health care services and others wanted to have family planning, maternal and child health, and nutrition combined.^27^^–^^29^ We focused on maternal care based on the concept of the continuum of care framework using essential maternal services during pregnancy, intrapartum, and postpartum periods. The calculation used 9 indicators of essential interventions, 5 for antenatal, 2 for intrapartum, and 2 for postnatal care. The indicator score is calculated on a scale between 0–100; the ideal score is the maximum attainable score (utilization rate), which is 100; the target is the ideal performance of the indicator. For our analysis, we calculated the mean score of maternal care service utilization at the individual level. The overall mean score is considered as a cut-off point to have dichotomous outcome variables for the logistic regression analysis.

$$Maternal\;care\;C=\frac{\frac{\left(ANC4\;+\;EANC\;+\;TT\;+\;HIVtest\;+\;FeFol\right)}{5}+\frac{\left(SBA\;+\;HFdel\right)}{2}+\frac{\left(PNC\;+\;EPNC\right)\;}{2}}{3}$$

where ANC4 is at least 4 ANC visits, EANC is initiated early ANC visit (in the first trimester), TT is received TT vaccination, HIVtest is tested for HIV (as part of PMTCT), FeFol is received iron folic acid supplement, SBA is skilled birth attendance, HFdel is delivered at health facility, PNC is have postnatal checkup, and EPNC is received early postnatal care (in the first 24 hours).

Potential explanatory variables at the individual level include residence, religion, marital status of the mother, educational status of the mother and partner/husband, main occupation of the mother/partner, wealth index (computed from 10 asset variables), and household distance from health facilities (in kilometers). Similarly, 4 categories of facility-level HIS variables (from the facility survey), including HIS infrastructure, data quality, data use, and data management, were used for explanatory variables. Details of the operational definitions are included in the Supplement.

Most of the facility-level variables were composite indices with averaged scores. Because of the study design, the nature of the data requires accounting for clustering at the health facility level as well as controlling all potential confounders at different levels (both at the health facility and individual service user levels). A series of modeling procedures was applied, starting from the null model to a more complex model involving individual and facility-level variables. After running the null model, the intraclass correlation coefficient (ICC) was estimated to know the percentage of total variation explained by variation between health facilities. Finally, individual and group level variables were checked and entered to produce the last model.

### Ethics Approval

Ethical clearance was secured from the Institutional Review Board of the Ethiopian Public Health Association. Support letters from the MOH, regional health bureaus, and woreda health offices were secured. A written consent form explaining the purpose and benefit of the study, participants’ right to withdraw at any point of the data collection process, confidentiality, and other associated issues was given to study participants (mothers having children aged 12–23 months) to document their agreement to participate in the study.

## RESULTS

### Background Characteristics of Study Participants

A total of 3,016 households with targeted women and children were reached as planned. More than half (53%) of the participants were aged 20–30 years. One-third (33%) did not attend formal education. Only 5% of mothers were employed by the government or private institutions while their partners had a higher percentage (17.6%) (Table 1).

**TABLE 1.** Background Characteristics of Respondents, Baseline Survey of Ethiopia Maternal Health Care Service Utilization Study, 2020

	**N**	**No. (%)**
Residence (regional clusters)	3,016	
Emerging regions		580 (19.2)
Agrarian regions		2,030 (67.3)
Urban		406 (13.5)
Mother’s age, years (mean: 28.3 years)	2,988	
15–19		168 (5.6)
20–24		677 (22.7)
25–29		913 (30.6)
30–34		614 (20.6)
35–39		504 (16.9)
40–44		96 (3.2)
45–54		16 (0.5)
Child’s age, months	3,016	
12–17		1,622 (53.8)
18–23		1,394 (46.2)
Currently married	3,016	2832 (93.9)
Religion	3,016	
Orthodox Christian		1,288 (42.7)
Muslim		1,261 (41.8)
Protestant		424 (14.1)
Other		43 (1.4)
Mothers’ educational status	3,016	
No education		991 (32.9)
Elementary (1–8)		1,388 (46.0)
Secondary		469 (15.6)
Above secondary		168 (5.6)
Partner educational status	2,832	
No education		712 (25.1)
Elementary (1–8)		1,208 (42.7)
Secondary		599 (21.2)
Above secondary		313 (11.1)
Mother’s occupational status	3,016	
Employed (Government or private institution)		154 (5.1)
Farmer		864 (28.6)
Merchant		185 (6.1)
Unemployed		1,577 (52.3)
Other		236 (7.8)
Partner occupation status	2,832	
Employed (Government or private institution)		497 (17.6)
Farmer		1,480 (52.3)
Merchant		153 (5.4)
Unemployed		54 (1.9)
Others		648 (22.9)

### Maternal Health Care Service Utilization Indicators

#### Utilization of ANC Service

As Table 2 indicates, 93% of women attended ANC at least once during their pregnancy, with 61% initiating ANC within the first 4 months of pregnancy. Fifty-four percent of women attended at least 4 ANC visits with a skilled health provider during their pregnancy. Of those women who attended ANC, 82% reported taking iron and folic acid (IFA) supplementation during their pregnancy and 46% of these women took iron and folic acid for 3 or more months of their pregnancy. Among women who attended ANC, 85% received HIV testing and 86% received tetanus toxoid vaccinations.

**TABLE 2.** Utilization of ANC Service Among Women Having Child Aged 12–23 Months, Ethiopia Baseline Survey, 2020

	**N**	**No. (%)**	**95% CI**
At least 1 ANC visit with skilled provider	2,967	2,755 (92.9)	91.8, 93.7
No. ANC visits in last pregnancy	2,967		
0		212 (7.1)	6.3, 8.1
1–3		1,152 (38.8)	37.1, 40.6
4+		1,603 (54.0)	52.2, 55.8
Gestational age at first ANC visit	2,802		
1–4 months		1,709 (61.0)	59.2, 62.8
5–9 months		1,037 (37.0)	35.2, 38.8
I don’t know		56 (2.0)	
IFA supplements	2,967	2,434 (82.0)	80.6, 83.4
Length of IFA use	2,967		
0–29 days		322 (10.9)	9.8, 12.0
30–59 days		667 (22.5)	21.0, 24.0
60–89 days		603 (20.3)	18.9, 21.8
90+ days		1,375 (46.3)	44.6, 48.1
HIV test (PMTCT)	2,802	2,376 (84.8)	83.4, 86.1
TT vaccination	2,802	2,413 (86.1)	84.8, 87.3

**Abbreviations:** ANC, antenatal care; CI, confidence interval; TT, tetanus toxoid; IFA, iron folic acid; PMTCT, prevention of mother-to-child transmission of HIV.

#### Utilization of Delivery PNC and Maternal Health Care Services

Both delivery in a health facility and SBA were high at 76% (Table 3). Of those attended by an SBA, 61% of births were attended by nurses and/or midwives. Among women who reported home delivery, 36% reported that they had no time to go to the health facility and 25% reported they did not believe it was necessary to go for delivery (data not shown). Of all reported births, 9% were cesarean deliveries.

**TABLE 3.** Utilization of Delivery, PNC, and Maternal Health Care Service Among Women Having Child 12–23 Months, Ethiopia Baseline Survey, 2020

		**No. (%)**	**95% CI**
Preterm births		43 (1.4)	1.0, 1.9
Delivery (n=2967)			
Delivered at health facility		2,249 (75.8)	74.2, 77.3
Delivered at home or in community		718 (24.2)	22.7, 25.8
Skilled birth attendance		2,248 (75.8)	74.2, 77.3
Cesarean delivery		271 (9.1)	8.1, 10.2
Payment for delivery service		447 (15.1)	13.8, 16.4
Type of provider for delivery service			
Doctor		424 (14.3)	
Health officer		160 (5.4)	
Nurse/midwife		1,809 (61.0)	
TBA		198 (6.7)	
Health extension		23 (0.8)	
Relative		593 (20.0)	
Others		34 (1.2)	
Unknown		268 (9.0)	
PNC service from a skilled health provider		2,094 (70.6)	68.9, 72.2
Facing maternal complication		333 (11.2)	10.1, 12.4
Overall maternal service coverage composite indicator			
Mean		71.0	69.9, 72.1
Below mean		1,139 (38.4)	36.6, 40.2
Above mean		1,828 (61.6)	59.8, 63.3
	Total	2,967	

**Abbreviations:** CI, confidence interval; PNC, postnatal care; TBA, traditional birth attendant.

Of the 71% of mothers who received PNC services, 94% of PNC checkups were in governmental health facilities (hospitals or health centers); 55.8% received PNC service within the first 1–2 hours of delivery, and 32.1% received PNC within the first 3–24 hours of delivery. The mean of the overall maternal service composite indicator (antenatal, intrapartum, and postnatal) was 71% (Table 3).

The percentage of mothers who had maternal complications was 11% (95% confidence interval (CI)=10.1, 12.4) (Table 3). Bleeding (36%) and prolonged labor (22.2%) were the most common complications listed by respondents. Among women who had maternal complications, 86% received assistance at health facilities with half (48%) of these women using ambulances for transport to the facility during their maternal complications.

### HIS Resources Availability and HIS Performance

The average score of availability of key HIS infrastructure at the health facility level was 82%, electronic HIS (eHIS) tools availability score was 39%, and 72% of health facilities had at least 1 trained staff in any of the HIS topics (Table 4). Regarding data quality, skilled birth attendance source document completeness of health facilities and verification of the quality in completing primary source documents (e.g., registers and patient records) was analyzed for each of the 3 months (April, May, and June 2020). Accordingly, the percentage of health facilities with complete source documents for all 3 months was 73%. Report accuracy in the acceptable range was 74%. However, 6% and 20% of health facilities had under-reporting and over-reporting, respectively. The main reasons cited for the observed discrepancies between the recorded data and report include arithmetic error, data entry error, and lack of emphasis on data accuracy. The average score of health facilities using routine data to improve HMIS data quality was 45%, and the average score of health facilities using data for performance review and evidence-based decision making was 50%. Health facilities that use routine data for annual planning and target setting was 84%, and 31% had evidence of analytical report production using HMIS data (Table 4).

**TABLE 4.** HIS Resource Availability and Performance of Health Facilities Based on Selected HIS Indicators, Ethiopia, 2020

	**Score, %**	**95% CI**
**HIS resource**		
HIS infrastructure at facility level (average score)	82	81, 83
eHIS tools availability score	39	38, 40
Number of staff with training on HIS topics at facility level 1 year prior to survey (average score)	7.8	7.4, 8.1
Percentage of facilities with at least 1 trained staff in any HIS topics	72	70, 74
**Data management**		
Data quality control practices (average score)	72	71, 74
Level of data analysis practice (average score)	68	67, 69
Data visualization practice	86	84, 87
Presence of feedback mechanism	92	90, 93
**Data quality** ^a^		
Source document completeness: all three months complete	73	71, 75
Data accuracy: acceptable range (90%–110%)	74	72, 76
Data accuracy: overreporting (<90%)	20	19, 22
Data accuracy: underreporting (>110%)	6	5, 7
**Data use**		
Use of routine data for RHIS quality improvement (average score)	45	43, 46
Use of routine data for performance review and evidence-based decision making (average score)	50	48, 51
Use of data for annual plan and target setting	84	83, 85
Use of data to produce narrative analytical reports	31	29, 32

**Abbreviations:** CI, confidence interval; eHIS, electronic health information system; HIS, health information system; RHIS, routine health information system.

^a^ Data quality measures (source document completeness and accuracy calculation) based on 3 months of skilled birth attendance data (April, May, June 2020).

### Associations Between HIS Performance Status and Maternal Service Utilization

In our analysis, the linked survey design ends up with linked data. Variables from the household survey (individual level) and health facility survey (health facility level) were taken for estimating associations between HIS variables and maternal health care service utilization. ANC4+, SBA, and maternal care service coverage composite indicator were selected as outcome variables.

#### Factors Associated With ANC4+ Visits

The difference among health facilities explains 26% of the variation of having ANC4+ visits during pregnancy (intraclass correlation coefficient (ICC) (ANC4+): 0.26; 95% CI= 0.19, 0.34).

The odds of having 4 or more ANC visits is 3.4 times higher among mothers living in urban regions, compared with residents in the pastoralist regions (adjusted odds ratio [AOR]: 3.42; 95% CI=1.48, 7.87). Similarly, the odds of having 4 or more ANC visits is 2 times higher in mothers who achieved secondary and above education (AOR: 2.02; 95% CI=1.55, 2.65) and 29% higher in mothers who achieved primary education, compared with mothers with no education (AOR, 1.29; 95% CI: 1.06, 1.58). Mothers who belong to the highest wealth quantile received 4 or more ANC visits and was 65% higher compared with mothers who belong to the lowest wealth quantile (AOR: 1.65; 95%CI=1.09, 2.51) (Table 5). For each additional eHIS tools availability score of health facilities, the odds of having 4 or more ANC visits is higher by 33% in mothers who received care from such health facilities (AOR: 1.33; 95% CI=1.07, 1.65). For each additional score of SBA reporting accuracy, the odds of a mother’s ANC4+ visit is 4.3 times higher but has a wide CI (AOR: 4.3; 95% CI=1.24, 15.36).

**TABLE 5.** Factors Associated With ANC4+ Visits, Ethiopia Baseline Survey, 2020

	**AOR**	**P**	**95% CI**
Regional clusters (residence)			
Pastoralist	1		1
Agrarian	1.53	0.147	.86, 2.71
Urban	3.42	0.004	1.48, 7.87^a^
Educational status of mother			
No education	1		1
Elementary (1–8)	1.29	0.010	1.06, 1.58^a^
Secondary and above	2.02	0.000	1.55, 2.65^a^
Wealth index			
Lowest	1		1
Low	1.15	0.302	.88, 1.49
Middle	1.13	0.383	.86, 1.48
High	1.24	0.186	0.90, 1.72
Highest	1.65	0.018	1.09, 2.51^a^
HIS infrastructure availability score	1.07	0.271	.95, 1.21
eHIS tools availability score	1.33	0.009	1.07, 1.65^a^
SBA reporting accuracy score	4.36	0.022	1.24, 15.36^a^
_constant	0.05	0.000	0.01, 0.21

Abbreviations: ANC4+, 4 or more antenatal care visits; AOR, adjusted odds ratio; CI, confidence interval; eHIS, electronic health information system; HIS, health information system; SBA, skilled birth attendance.

^a^ Significantly associated.

#### Factors Associated With SBA Rate

Difference among health facilities explains 55% of the variation of SBA. Intraclass correlation coefficient (ICC=0.55; 95% CI=0.44, 0.65) (Table 6). The odds of SBA was about 2 times higher among mothers who achieved primary education (AOR: 1.90; 95% CI=1.48, 2.43) and 3.4 times higher among mothers who achieved secondary education or above (AOR: 3.43; 95% CI=2.26, 5.23), compared with mothers with no education. A significant and increasing trend in the association between wealth quintile and SBA was seen when the lowest quintile is used as reference. For each additional kilometer distance from the house to health facility, the odds of SBA decreased by 93% (AOR: 0.93; 95% CI=0.89, 0.97).

**TABLE 6.** Factors Associated With SBA Among Mothers, Ethiopia Baseline Survey, 2020

	**Odds Ratio**	**P>z**	**95% CI**
Belongs to HIS intervention woreda	1.53	0.42	0.54, 4.34
Regional clusters (residence)			
Pastoralist	1		1
Agrarian	1.17	0.774	0.40, 3.44
Urban	4.46	0.083	0.82, 24.19
Educational status of mother			
No education	1		1
Elementary (1–8)	1.90	0.000	1.48, 2.43^a^
Secondary and above	3.43	0.000	2.26, 5.23^a^
Wealth index			
Lowest	1		1
Low	1.41	0.031	1.03, 1.93^a^
Middle	1.80	0.000	1.29, 2.51^a^
High	2.45	0.000	1.56, 3.83^a^
Highest	5.64	0.000	2.52, 12.60^a^
SBA source document completeness			
Not complete	1		1
1 or 2 months complete	0.49	0.326	0.12, 2.01
All months complete	1.69	0.288	0.64, 4.44
Distance from HH to HF (km)	0.93	0.001	0.89, 0.97^a^
HIS infrastructure availability score	1.14	0.23	0.92, 1.43
_constant	0.77	0.765	0.14, 4.13

Abbreviations: CI, confidence interval; HF, health facility; HH, household; HIS, health information system; km, kilometer; SBA, skilled birth attendance.

^a^ Significantly associated.

#### Factors Associated With Maternal Care Service Composite Indicator

The difference among health facilities explains 31% of the variation in the maternal care service coverage (maternal care ICC: 0.31; 95% CI=0.23, 0.40). Education of mother, wealth index, eHIS availability score, and distance to health facilities (marginal) were significantly associated with maternal care service coverage (Table 7).

**TABLE 7.** Factors Associated With Maternal Care Service Composite Indicator, Ethiopia Baseline Survey, 2020

	**Odds Ratio**	**P>z**	**95% CI**
Educational status of mother			
No education	1		1
Elementary (1–8)	1.52	0.000	1.24, 1.86^a^
Secondary and above	2.74	0.000	2.04, 3.68^a^
Wealth index			
Lowest	1		1
Low	1.34	0.036	1.02, 1.80^a^
Middle	1.5	0.009	1.10, 2.0^a^
High	1.7	0.003	1.2, 2.4^a^
Highest	1.95	0.003	1.26, 3.02^a^
SBA source document completeness			
Not complete	1		1
1 or 2 months complete	0.68	0.42	0.27, 1.73
All months complete	1.60	0.14	0.86, 2.96
Distance from HH to HF (per additional km)	0.96	0.05	0.93, 0.99^a^
eHIS tools availability score	1.43	0.003	1.13, 1.82^a^
_constant	0.44	0.033	0.20, 0.94

Abbreviations: CI, confidence interval; eHIS, electronic health information system; HF, health facility; HH, household; km, kilometer; SBA, skilled birth attendance.

^a^ Significantly associated.

## DISCUSSION

This study revealed that maternal care service coverage indicators are much higher compared to reports from Ethiopia DHS and other population-based surveys.^5^ This may be due to the government’s and its partners’ commitments to improve access to maternal, newborn, and child health services in recent years.^7^^,^^9^ Monitoring of such key public health programs is not possible without a functional HIS that has high-quality data and is used for decision making. HIS interventions in the project sites are expected to continue to help improve HIS performance. We believe that health program managers used the intervention as an opportunity to sustain the current utilization rate and improve the quality of service.

This study revealed that maternal care service coverage indicators are much higher compared to reports from Ethiopia DHS and other population-based surveys.

ANC service coverage is very high; however, there are gaps in visit quality and frequency. For example, a significant number of mothers did not receive the essential interventions included within the ANC service package and nearly half of these women had less than 4 ANC visits, which is below the recommended standard. Similarly, only 60% of these women initiated ANC within the first 4 months of pregnancy. This is in line with findings from similar studies.^17^^,^^18^ The findings imply that improving the quality of care by ensuring the availability of essential services in the ANC service package and using strategies to increase ANC4+ are mandatory.

Coverage of SBA, delivery in a health facility, and PNC were much higher compared to previous studies. For example, the current SBA (75%) is significantly higher than the Mini Ethiopia DHS report (50%).^5^ This difference could be linked to program interventions implemented throughout the country and due to the study population difference; involving study subjects from a recent time period, limited study area (project area and controls), and denominator restricted to mothers with a child aged 12–23 months. One of the most critical interventions identified for maternal and child survival is ensuring that a health worker with midwifery skills is present at every birth since most obstetric complications are difficult to predict, and any woman can suddenly develop a life-threatening emergency.^1^ Providing payment-free delivery service is one of the government’s commitments to minimizing barriers to access, but mothers were obliged to pay in all private and even in some government health facilities.

Providing timely and lifesaving care for maternal complications is another priority for maternal and child survival. Eleven percent of women faced pregnancy-related complications, and 86% of these women received assistance at health facilities. Bleeding was the most frequently reported complication among these women, which is also reported as a major cause of maternal death in many studies.^30^ Nine percent of women delivered by cesarean delivery, which is in line with the reported pregnancy-related complications and within the recommended range of 5%–15%.^31^

HIS performance indicators were measured to see the current level of performance and their association with the uptake of maternal services. The study showed that the HIS resource availability scores of health facilities like HIS infrastructure (82%) are good. However, eHIS tools availability score (39%) is very low. Electronic and digital systems allow health facility staff to collect some types of routine health information faster and with fewer mistakes.^32^ The finding implies that interventions are needed to improve the availability of functional eHIS tools.

The study highlights that most of the data quality and data use indicators showed suboptimal performance compared with the national target of 90%. The finding is consistent with previous studies in Ethiopia, which indicated that data quality and use of routine health information by health workers and health managers is low.^24^^,^^33^^,^^34^ Based on a systematic literature review, researchers suggested that interventions facilitating data availability combined with technology enhancement increased the use of data. Combinations of technology enhancement along with capacity-building activities, and data quality assessment and feedback system were recommended for improving data quality.^35^ Conducting routine data quality assessments and implementing interventions based on the identified gaps can help improve data quality.^36^^,^^37^

The study highlights that most of the data quality and data use indicators showed suboptimal performance compared with the national target of 90%.

The multilevel analysis showed that HIS indicators, such as the availability of eHIS tools and data accuracy, were significantly associated with ANC4+. The availability of eHIS tools is also significantly associated with the maternal care composite indicator. This finding implies that decisions based on quality data are crucial for improving maternal service uptake. The presence of eHIS tools, such as a functional DHIS2 system and electronic medical record in health facilities, are key factors in producing quality data and encouraging information use. Having a user-friendly and acceptable HIS is mandatory for the management and use of routine data. Health workers and HIS technicians should have information technology and systems to use data and improve service quality.^38^ To strengthen analytic capacity and use of eHIS tools, need-based trainings should be planned and provided.

Individual-level variables such as maternal education, wealth index, and distance from health facilities, were significantly associated with coverage indicators. The role of maternal education in maternal service utilization has been observed in many studies within Ethiopia and elsewhere.^11^^,^^39^^,^^40^ The disparity of maternal service utilization by wealth index has also been well- established in other studies.^10^^,^^12^ The negative effect of distance for SBA or maternal care at the health facility is visible in this study and others.^2^ Labor or delivery is an emergency that requires reachable health facilities as quickly as possible. This issue of proximity could be addressed by having nearby health facilities or a functional transportation system. In our context, many mothers will not have access to an ambulance in an emergency situation. Ensuring accessibility of health facilities within a reasonable distance should be a priority for delivery care service in Ethiopia.

### Limitations

The findings of the study should be interpreted by considering the following limitations. First, the current study uses project sites. Although all regions in Ethiopia were included, the study findings can be generalizable only to the study sites. Second, service coverage for maternal health care was measured at least 1 year ago. Therefore, the assumption of no change in health facility measurements of HIS performance for the past year or more may not work if HIS interventions were intensified during this period. Moreover, mothers may have difficulties recalling some of the events that happened 1 or 2 years ago. Third, the analysis did not see the influence of other facility-level variables such as availability of trained staff and essential medicines and supplies, which may correlate with HIS strength and underlie the associations detected in this analysis. Fourth, the impact of the coronavirus disease (COVID-19) pandemic was not controlled. The study used 3-month documentation and report evaluations after the height of the pandemic in Ethiopia, which may have had an impact on documentation and reporting. Fifth, in most composite indices, calculations were done based on a series of questions with binary outcomes. These questions have usually provided equal weight with “0” for “no/absent” responses and “1” for “yes/present” responses. The provision of “equal weight” may not necessarily reflect the contributions of certain measurements. Finally, because of the cross-sectional design, some associations cannot be free from reverse causality.

## CONCLUSION

In conclusion, most of the maternal service coverages, including ANC services, SBA, cesarean delivery, and PNC, showed promising performance. However, there were many issues that will require program-level interventions. HIS performance is suboptimal. Individual-level variables, including mother’s education status, wealth index, and distance to the health facility, were significantly associated with most of the maternal service indices. From the HIS performance indices, data accuracy and availability of eHIS tools were common variables significantly associated with maternal health care service utilization. Maintaining the current coverage and improving the quality of care will be a major challenge that requires attention from program coordinators and health workers. Offering need-based trainings, availing guidelines, planning motivation strategies to have better documentation and reporting practices by health professionals and HIS workers, and strengthening data quality control practices are among the crucial interventions to improve HIS performance and thereby improve maternal service quality and uptake. Conducting similar research outside of the project sites will be helpful to gain a wider understanding of HIS data quality and information use across Ethiopia.

## Supplementary Material

GHSP-D-21-00688-supplement.pdf

## Notes

**Peer Reviewed**
